# Occurrence of *Alternaria* and Other Toxins in Cereal Grains Intended for Animal Feeding Collected in Slovenia: A Three-Year Study

**DOI:** 10.3390/toxins13050304

**Published:** 2021-04-24

**Authors:** Janja Babič, Gabrijela Tavčar-Kalcher, Franci Aco Celar, Katarina Kos, Tanja Knific, Breda Jakovac-Strajn

**Affiliations:** 1 Institute of Food Safety, Feed and Environment, Veterinary Faculty, University of Ljubljana, Gerbičeva 60, 1000 Ljubljana, Slovenia; janja.babic@vf.uni-lj.si (J.B.); tanja.knific@vf.uni-lj.si (T.K.); breda.jakovacstrajn@vf.uni-lj.si (B.J.-S.); 2 Department of Agronomy, Biotechnical Faculty, University of Ljubljana, Jamnikarjeva 101, 1000 Ljubljana, Slovenia; franc.celar@bf.uni-lj.si (F.A.C.); katarina.kos@bf.uni-lj.si (K.K.)

**Keywords:** mycotoxins, *Alternaria*, liquid chromatography–tandem mass spectrometry (LC–MS/MS), occurrence, co-occurrence, cereal grains

## Abstract

In recent years, the less-studied *Alternaria* mycotoxins have attracted increasing interest due to the lack of survey data and their ability to cause toxic effects in animals and humans. To fill the gap, the aim of this three-year survey was to investigate the presence and co-occurrence of *Alternaria* and other mycotoxins in a total of 433 cereal grain samples from Slovenian farms and agricultural cooperatives from 2014 to 2016. Using the multi-mycotoxin method, 14 mycotoxins were determined. In 53% of 433 analysed samples, contamination with at least one mycotoxin was found. Deoxynivalenol (DON) and tenuazonic acid (TeA) were present in 32% and 26% of cereal grain samples, respectively, whereas alternariol (AOH), tentoxin (TEN), alternariol monomethyl ether (AME), 3- and 15-acetyldeoxynivalenol (3- and 15-AcDON), and zearalenone (ZEN) were present in fewer than 15% of the samples. Ochratoxin A (OTA) was found in one rye sample, while diacetoxyscirpenol (DAS), HT-2 and T-2 toxin, and fumonisins B_1_ and B_2_ (FB1 and FB2) were not detected. The highest maximum and median concentrations of *Alternaria* toxins were determined in spelt in 2016 (TeA, 2277 µg/kg and 203 µg/kg, respectively), and those of *Fusarium* toxins in wheat in 2015 (DON, 4082 µg/kg and 387 µg/kg, respectively). The co-occurrence of two or more mycotoxins was found in 43% of the positive samples. The correlations between *Alternaria* toxins were very weak but statistically significant (r: 0.15–0.17, *p*: 0.0042–0.0165). A well-known correlation between *Fusarium* toxins DON and ZEN was weak and highly significant (r = 0.28, *p* < 0.0001).

## 1. Introduction

Mycotoxins are secondary fungal metabolites of low molecular weight and can contaminate a wide range of food and feed commodities. *Aspergillus*, *Penicillium*, *Fusarium*, and *Alternaria* species have the potential to produce various mycotoxins in the field or during the storage of commodities due to poor storage conditions. Even with good agricultural, storage, and processing practices, contamination with mycotoxins is considered unavoidable. Exposure to these compounds is a significant threat to human and animal health and is associated with different acute or chronic mycotoxicoses [1,2,3]. Most mycotoxins are chemically and thermally stable during food processing, including boiling, cooking, baking, frying, and pasteurization [3,4,5,6,7,8]. Therefore, prevention of mycotoxin contamination in the field before harvest, during harvest, and during storage of commodities is one of the main objectives of the food and feed industry [8,9]. Consumption of feed contaminated with mycotoxins can lead to feed refusal, reduced feed conversion, and thus to reduced body weight, impairment of the immune system, and reproductive disorders. Mycotoxins present in products from animals fed contaminated feed (such as meat, eggs, and milk) can be consumed by humans [2,8,10,11].

Currently, more than 300 different mycotoxins are known [12]. However, only a limited number of mycotoxins are relevant for the food and feed industry. Most studies focus on mycotoxins that are regularly tested, namely aflatoxins (AFs), deoxynivalenol (DON), zearalenone (ZEN), fumonisins B_1_ and B_2_ (FB1 and FB2), and ochratoxin A (OTA). The presence of these toxins in feed is regulated by Commission Regulation No 574/2011 [13], amending Commission Directive 2002/32/EC [14], and by Commission Recommendation 2006/576 [15]. In recent years, the less-studied *Alternaria* mycotoxins have gained increasing interest due to their potentially toxic effects on human and animal health [1,16]. The genus *Alternaria* is ubiquitous and includes both plant pathogenic species that can damage crops in the field (e.g., maize, wheat, triticale, barley, oats, rye, spelt, etc.) and saprophytic species that can cause spoilage of plant products after harvesting [17,18]. *Alternaria* species produce about 70 different mycotoxins, but the most important mycotoxins are tenuazonic acid (TeA), tentoxin (TEN), alternariol monomethyl ether (AME), alternariol (AOH), and altenuene (ALT) [1,19]. The major *Alternaria* toxins include derivatives of dibenzo-α-pyrones (AOH, AME, ALT) and of tetramic acid (TeA), and other chemical structures such as the cyclic tetrapeptide (TEN). So far, little is known about their toxic properties and toxicological mechanisms, bioavailability, and stability in the digestive tract. *Alternaria* toxins have a harmful effect in animals, including cytotoxicity, fetotoxicity, and teratoxicity [20,21,22]. TeA is the most acutely toxic molecule in the group of *Alternaria* toxins [19,20]. Its toxicity has been reported in chick embryos and several animal species, including poultry, pigs, mice, rabbits, dogs, and monkeys [1,21,22]. Yekeler et al. [23] presented evidence for the involvement of AME and TeA in precancerous changes observed in the esophageal mucosa of mice. AOH and AME were reported to have genotoxic, mutagenic, and carcinogenic properties. AME and AOH induce DNA strand breaks and gene mutations in cultured human and animal cells [22].

Reports of the presence of *Alternaria* toxins in feed are rare. No contamination with TeA and AME was found in 129 wheat samples from the Czech Republic, but the presence of AOH and ALT was reported in 46.5% and 91.5% of samples, respectively [24]. In a study from Sweden, only 18 barley, wheat, and oat samples were analysed. AOH, AME, and TeA were found in 89%, 39%, and 100% of the samples, respectively. The maximum concentrations of AOH, AME, and TeA were 335 µg/kg, 184 µg/kg, and 4310 µg/kg, respectively [25]. In 22 Chinese wheat samples, AOH, AME, and TeA were present in 90.9%, 95.5%, and 100% of the samples, respectively. The highest contamination of the wheat samples was with TeA (6432 µg/kg) [26]. Azcarate et al. [27] reported contamination with TeA, AOH, and AME at rates of 72%, 87%, and 91%, respectively, and a maximum TeA concentration of 8814 µg/kg. *Alternaria* toxins were also determined in German wheat, which was mostly (30%) contaminated with TeA and, in less than 10%, with AME, AOH, and ALT [28]. In a Norwegian study [29], high concentrations of AOH, AME, and TeA were detected in 20 barley, 28 oat, and 28 wheat samples. The maximum concentration of AOH and AME was determined in oats (449 µg/kg and 177 µg/kg, respectively), and the maximum concentration of TeA in barley samples (247 µg/kg). Unlike reports on other mycotoxins from the Balkan region, data on *Alternaria* mycotoxins are missing, with the exception of a publication from Serbia by Janić Hajnal et al. [30] and a publication on *Alternaria* toxins in grains from Albania by Topi et al. [31]. Kirinčič et al. [32] reported the results of official controls on the presence of mycotoxins in cereals and cereal products for human consumption in Slovenia in 2008–2012. The occurrence of relevant mycotoxins in 107 samples from different grains intended for feed in 2007–2008 was reported by Jakovac-Strajn et al. [33], while no data on the occurrence of *Alternaria* toxins were reported for Slovenia so far. Currently, there are no regulations on *Alternaria* toxins in food and feed in Europe [1,16], but the presence of these toxins can lead to economic losses due to harvest and postharvest spoilage [3,34,35], and can affect human and animal health [1,16]. As European Food Safety Authority (EFSA) [1,16] recommended generating more analytical data on the occurrence of *Alternaria* toxins, the aim of our survey was to focus on *Alternaria* toxins (TeA, TEN, AOH, and AME) in cereal grains intended for animal feeding collected in Slovenia in 2014–2016. Additionally, *Fusarium* toxins (DON, 3-AcDON, 15-AcDON, DAS, HT-2, T-2, FB1, FB2, ZEN) and OTA were reported.

## 2. Results

### 2.1. Method Validation Data

LOD, LOQ, and correlation coefficients in the tested concentration range are given in Table 1. The presence of matrix components can affect the ionization of a target compound; furthermore, this effect is strongly dependent on the mycotoxin and the matrix. Therefore, a matrix-matched calibration was used to compensate the matrix effect. Good linearity was proven for all analytes, with correlation coefficients being higher than 0.9914.

The recoveries, repeatability (RSD_r_), and within-laboratory reproducibility (RSD_wR_) are presented in Table 2. The average recovery rate for single mycotoxins varied between 90% and 110%. The RSD_r_ ranged from 4.5% to 18%, while RSD_wR_ for DON, 3-AcDON, 15-AcDON, DAS, ZEN, OTA, HT-2, T-2, FB1, and FB2 was 11%–24% at LOQ, 8%–26% at 2LOQ, and 2%–22% at 20 times LOQ. For *Alternaria* toxins, RDR_wR_ were 15%–21% at LOQ, 9%–17% at 4LOQ, and 9%–20% at 40 times LOQ. The recoveries as well as the repeatability and within-laboratory reproducibility met the requirements of Commission Regulation 401/2006 [36]. The determined average recovery rates were used for the correction of results. The samples where the results exceeded the maximum validated concentration were diluted after extraction to fit the calibration range and measured again. The results were calculated considering the dilution factor.

### 2.2. The Occurrence of Mycotoxins by Year

**Figure 1:**
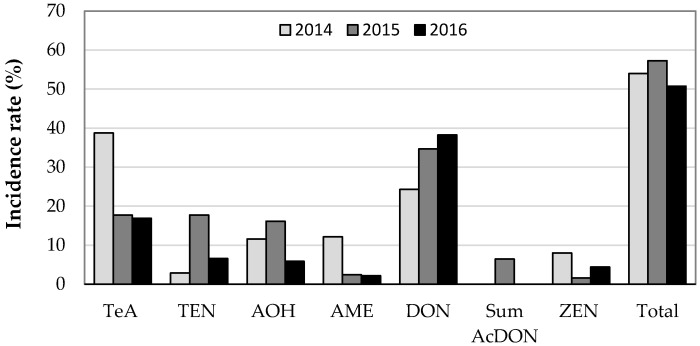
Incidence rate (%) of *Alternaria* and *Fusarium* mycotoxins by year: 2014 (No. = 173), 2015 (No. = 124), and 2016 (No. = 136).

**Figure 2:**
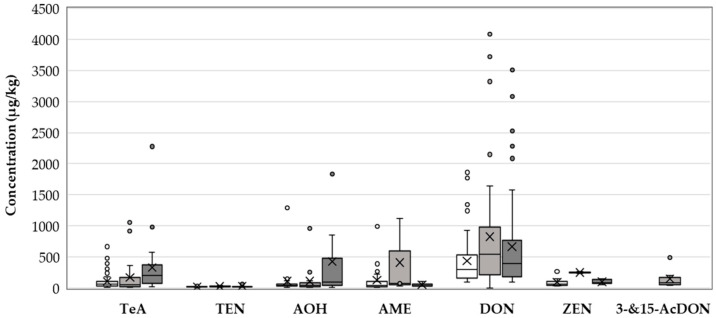
Concentrations of *Alternaria* toxins and *Fusarium* toxins by year. Median concentrations are indicated by horizontal lines in the boxes encompassing the 25th–75th percentiles. Whiskers indicate the 90th and 10th percentiles and outliers in the 95th and 5th percentiles are indicated by dots. Mean concentrations are indicated by ×. Results by year are indicated: 2014 (white), 2015 (light grey), and 2016 (dark grey).

A total of 433 ground cereal samples were analysed in the three-year survey; 173, 124, and 136 samples were analysed in 2014, 2015, and 2016, respectively. The samples in the study that contained one or more mycotoxins in concentrations equal to or higher than LOQ (Table 1) were considered positive. The results are shown in Figure 1 and Figure 2, and Supplementary Table S1. In total, 53% of samples were contaminated with one or more mycotoxins. The mycotoxins were present in 54%, 57%, and 51% of the tested samples in 2014 to 2016, respectively (Figure 1).

Most samples contained DON and TeA (32% and 26%, respectively). Furthermore, 11% of samples were contaminated with AOH, 8% with TEN, 6% with AME, 5% with ZEN, and 2% with DON metabolites 3-AcDON and 15-AcDON. OTA was determined in only one rye sample harvested in 2014 and the other mycotoxins (DAS, HT-2, T-2, FB1, and FB2) were not detected in the analysed samples. The mycotoxin concentrations ranged from 13 µg/kg to 4082 µg/kg. The total mean concentration of positive samples was 535 µg/kg and the median value was 275 µg/kg.

**Figure 3:**
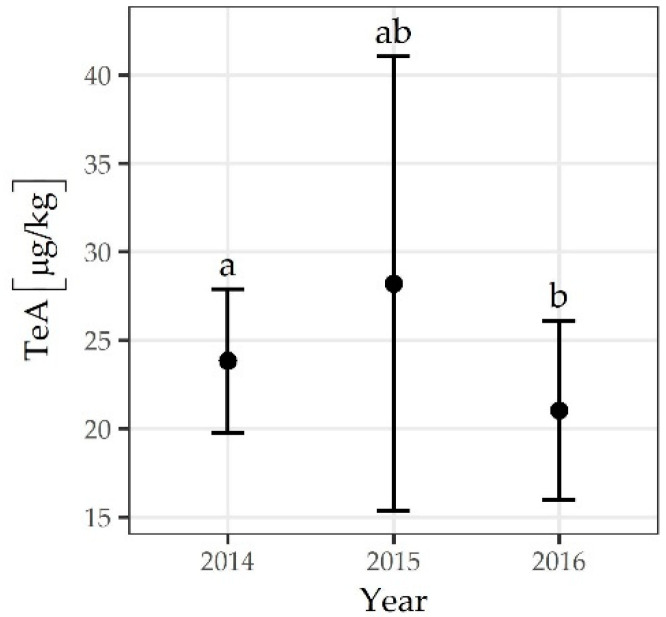
Mean concentration and standard error of TeA by year. The letters above the error bar (a, b) indicate the results of multiple comparison tests; groups with different letters are significantly different (*p* < 0.001).

The incidence of TeA was highest in 2014 (39%) (Figure 1), but the maximum concentration was found in 2016 (2277 µg/kg), with a mean concentration of 359 µg/kg and a median concentration of 219 µg/kg (Figure 2). The mean concentration of TeA in 2014 was significantly different from that in 2016 (*p* = 0.0003) (Figure 3). The contamination with AME was highest in 2014 (12%), whereas the highest maximum and median concentrations were determined in 2015 (1121 µg/kg and 78 µg/kg, respectively). The incidence rate of samples contaminated by TEN and AOH was highest in 2015, while maximum concentrations were found in 2016.

The incidence rate of DON was highest in 2016 (38%) and lowest in 2014 (24%). The highest maximum concentration for DON was determined in 2015 (4082 µg/kg), when the mean and median concentration in the positive samples were 846 µg/kg and 604 µg/kg, respectively (Figure 1). The metabolites 3-AcDON and 15-AcDON were not found in analysed samples in 2014 and 2016, whereas in 2015, 6% of samples contained 3-AcDON and 15-AcDON (Figure 1).

In 2014–2016, a total of 5% of the samples were contaminated with ZEN. The occurrence in 2014 and 2016 was 8% and 4% (Figure 1), respectively, whereas in 2015 only two samples contained ZEN. The maximum concentration was 300 µg/kg.

### 2.3. The Occurrence of Mycotoxins by Cereal Grain

**Figure 4:**
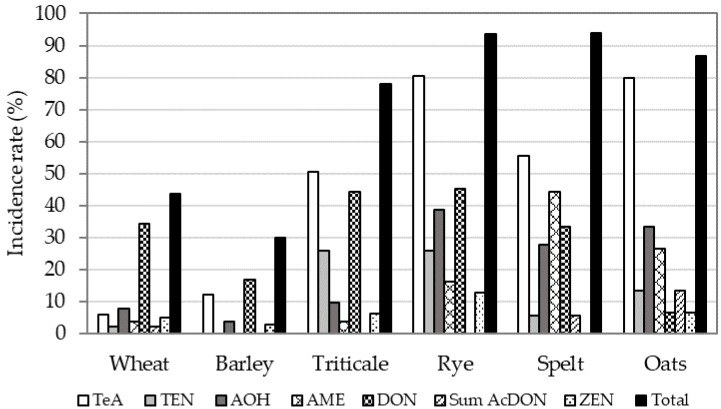
Incidence rate (%) of *Alternaria* and *Fusarium* mycotoxins in cereals: wheat (No. = 181), barley (No. = 107), triticale (No. = 81), rye (No. = 31), spelt (No. = 18), and oats (No. = 15).

**Figure 5:**
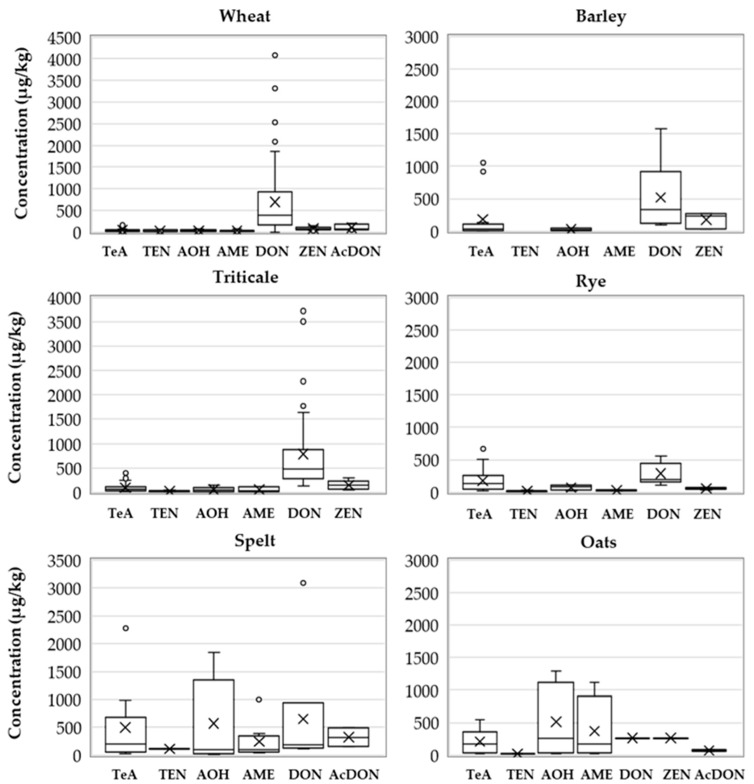
Concentrations of *Alternaria* toxins and *Fusarium* toxins in cereals. Median concentrations are indicated by horizontal lines in the boxes encompassing the 25th–75th percentiles. Whiskers indicate the 90th and 10th percentiles and outliers in the 95th and 5th percentiles are indicated by dots. Mean concentrations are indicated by ×.

The results are presented in Figure 4 and Figure 5, and Supplementary Table S2. The most contaminated cereals during the period of investigation were rye (94%), spelt (94%), oats (87%), and triticale (78%), while the contamination rates for wheat and barley were lower (44% and 30%, respectively). The mycotoxin pattern was different among various cereals (Figure 4).

The highest maximum concentration was found for DON in wheat (4082 µg/kg), although high maximum concentrations of DON were also found in triticale (3720 µg/kg) and spelt (3084 µg/kg). The median concentration in triticale samples (471 µg/kg) was higher than in wheat. Since almost all rye, oat, and spelt samples were contaminated with mycotoxins, high mean and median values were observed (Figure 5).

The wheat samples were less contaminated with DON metabolites (2%) and ZEN (5%). The maximum concentration for ZEN was 149 µg/kg with a median concentration of 74 µg/kg. Contamination of wheat samples with *Alternaria* toxins was less than 10%.

The mycotoxin incidence rates in triticale samples decreased in the following order: TeA (51%) > DON (44%) > TEN (26%) > AOH (10%) > ZEN (6%) > AME (4%) (Figure 4). The maximum concentration of DON in triticale was 3720 µg/kg with a median concentration of 471 µg/kg (Figure 5). The maximum concentration for TeA in triticale was 397 µg/kg, for TEN 42 µg/kg, and for AOH and AME 150 µg/kg and 115 µg/kg, respectively. The median concentration for *Alternaria* toxins was highest for TeA (78 µg/kg) and for AOH (46 µg/kg), while for TEN and AME they were 25 µg/kg and 23 µg/kg, respectively (Figure 5).

**Figure 6:**
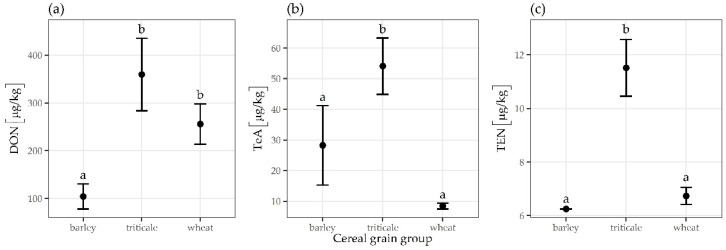
Mean concentrations and standard errors of the mean for (**a**) DON, (**b**) TeA, and (**c**) TEN by cereals. Letters above the error bars (a, b) indicate the results of multiple comparison tests; groups with different letters are significantly different (*p* < 0.001).

Barley samples were contaminated with DON (17%), TeA (12%), AOH (4%), and ZEN (3%). However, the maximum concentration of DON and TeA was 1573 µg/kg and 1053 µg/kg, respectively. Contamination with DON was significantly lower in barley than in triticale and wheat (*p* = 0.0003), and contamination with TeA and TEN was significantly higher in triticale than in barley and wheat (*p* < 0.001) (Figure 6).

In the samples of rye, spelt, and oats, contamination with *Alternaria* toxins was higher than with *Fusarium* toxins (Figure 5). All four *Alternaria* toxins were detected in these cereals, and contamination with TeA was more frequent than with other *Alternaria* toxins. In spelt, the maximum concentration of DON, TeA, and AOH was 3084 µg/kg, 2277 µg/kg, and 1836 µg/kg, respectively. DON metabolites were found in 11% of spelt samples. ZEN was present in 13% and 7% of rye and oat samples, respectively, while it was not detected in spelt samples.

### 2.4. Co-Occurrence of Mycotoxins in Cereal Grains in 2014–2016

**Figure 7:**
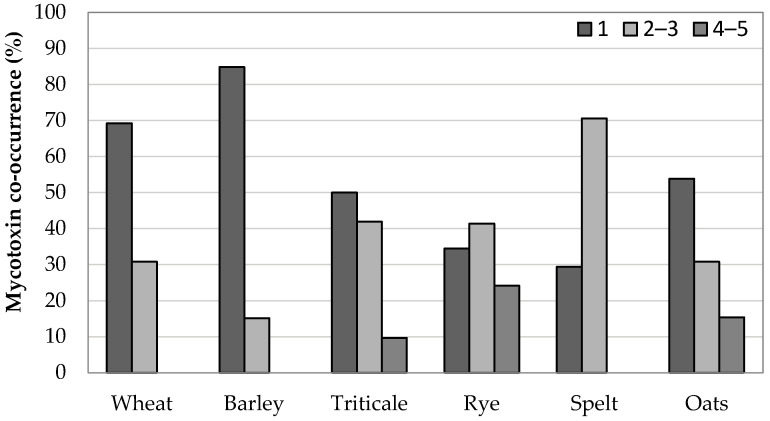
Co-occurrence of mycotoxins in a total of 231 positive samples of various cereals: wheat (No. = 78), barley (No. = 33), triticale (No. = 62), rye (No. = 29), spelt (No. = 17), and oats (No. = 13).

In all the years studied, the contaminated samples mainly contained one mycotoxin (58%). The co-occurrence of two, three, four, and five mycotoxins was detected in 26%, 11%, 5%, and 1% of the positive samples, respectively. Figure 7 shows the co-occurrence of mycotoxins in individual cereals. In wheat and barley, positive samples containing one mycotoxin were by far the most common. Triticale and oat samples also most frequently contained one mycotoxin. However, many samples contained two or three mycotoxins. On the other hand, positive rye and spelt samples mainly contained more than one mycotoxin.

In 78 positive wheat samples, the most frequent contamination was with DON, which co-occurred with ZEN (11%) and its metabolites 3-AcDON and 15-AcDON (6%). The co-occurrence rate of DON with one or more *Alternaria* toxins was 22%. Concerning the *Alternaria* toxins, the co-occurrence of pairs AOH–AME, TEN–AOH, and TeA–AME was found in wheat samples. Positive barley samples were mainly contaminated with DON, which co-occurred in 8% of cases with ZEN, while the co-occurrence with its metabolites was not found. The co-occurrence of TeA and AOH was found in three samples. 

The co-occurrence of the pair DON–ZEN in triticale samples was 8%. DON and at least one *Alternaria* mycotoxin co-occurred in 66% of the positive samples. In 15%, 11%, 5%, and 5% of triticale positive samples, co-occurrence of *Alternaria* toxin pairs TeA–TEN, TeA–AOH, TeA–AME, and AOH–AME was found. In rye and oat samples, the co-occurrence of DON and ZEN was 14% and 8%, respectively. The most common co-occurrence pairs of *Alternaria* toxins in rye, spelt, and oat samples were TeA–AOH, TeA–AME, TeA–TEN, and AOH–AME.

Overall, in all three years, the most frequently co-occurring *Alternaria* toxins were TeA–AOH (10%), TeA–TEN (8%), TEN–AME (7%), and AOH–AME (5%), while the other co-occurrences were below 3%. Among *Fusarium* mycotoxins, DON–ZEN appeared together in 9% of the positive samples, while DON and its metabolites co-occurred in less than 5% of positive samples.

Significant positive correlations were found in the concentrations between several pairs of mycotoxins. The correlations among *Alternaria* toxins TeA and AME, AOH, and TEN were very weak but statistically significant (r: 0.15–0.17, *p*: 0.0042–0.0165). The correlation between AME and AOH was weak but highly significant (r = 0.22, *p* < 0.0001). A well-known correlation between *Fusarium* toxins DON and ZEN in feed [37,38,39] was found in the study. It was weak and highly significant (r = 0.28, *p* < 0.0001). The only moderate correlation was found between DON metabolites 3-AcDON and 15-AcDON (r = 0.71, *p* < 0.0001).

## 3. Discussion

In 2009, Jakovac-Strajn et al. [40] investigated the presence of toxigenic moulds of the genera *Fusarium*, *Penicillium*, *Aspergillus*, and *Alternaria* in cereals grown in Slovenia. *Fusarium* spp. were the most widespread, but moulds of the genus *Alternaria* spp. were also found in 44% of the investigated samples and were identified as a potential risk of contamination of animal feed with *Alternaria* toxins. The results of the present study proved the presence of one or more *Alternaria* toxins in cereals used in Slovenia (Figure 1 and Figure 4). *Alternaria* toxins were present in all cereals. The results show that the number of positive samples for all four *Alternaria* toxins, TeA, TEN, AOH, and AME, was highest for rye, spelt, oats, and triticale. In accordance with the results of published studies [25,26,28,29,30], our study also found that TeA is the most common *Alternaria* toxin in cereals. It appeared in 85%, 48%, and 70% of *Alternaria* positive samples in 2014, 2015, and 2016, respectively. TEN appeared in 6%, 48%, and 27% of *Alternaria* positive samples in these years, respectively, AOH in 25%, 43%, and 24% of *Alternaria* positive samples and AME in 27%, 7%, and 9% of *Alternaria* positive samples. As the ratio between *Alternaria* toxins is not constant over the investigated years, no conclusions on *Alternaria* toxin pattern can be drawn. In the study on wheat from Serbia conducted by Janić Hajnal et al. [30], 68.5% of the samples contained TeA, 12.5% AOH, and 6.5% AME, while in our results wheat was mostly contaminated with DON and below 10% with *Alternaria* toxins. The AOH and AME mean values in the cereals obtained in this study (156 µg/kg and 153 µg/kg) were higher than the mean values in Serbian wheat reported by Janić Hajnal et al. [30] and the mean values in cereals (wheat, oats, spelt, and rye) in the EFSA report [1]. The highest maximum concentration of TeA, TEN, and AOH was found in spelt (2277 µg/kg, 116 µg/kg, and 1836 µg/kg, respectively) and the highest concentration of AME was found in oats (1121 µg/kg). Uhlig et al. [29] reported higher TeA concentrations in barley samples than in wheat and oat samples. In our study, the observed occurrence of *Alternaria* toxins in cereals (Figure 1 and Figure 4) was higher than in the EFSA *Alternaria* toxin opinion [1], where the occurrence of the four *Alternaria* toxins in cereal samples was 6% (TeA), 3% (AOH), 2% (AME), and 3% (TEN). On the other hand, some studies [23,24,25] showed high contamination rates with AOH, AME, ALT, and TeA in cereal samples, ranging from 39% to 100%.

Cereals were most frequently contaminated with DON (32%). The number of DON positive samples increased slightly each year. In 2014, 2015, and 2016, the contamination rate was 24%, 35%, and 38%, respectively. A similar contamination with DON was found in wheat samples in Southern Europe (38%), Romania (43%), Italy (28%), and Albania (23%) [41,42,43,44]. A higher incidence of DON in wheat was reported from the Mediterranean countries (Spain, Tunisia), where the contamination was 75% and 83%, respectively [45,46]. The maximum and mean concentration of DON in wheat was 4082 µg/kg and 387 µg/kg, respectively. Similar data for the mean and maximum concentration of DON in wheat and other cereals were reported in the previously mentioned work of Rodrigues and Naehrer [41], Streit et al. [11], and EFSA [47]. The maximum value for DON is comparable to the maximum values for wheat samples from Southern Europe, South America, and North Asia, as reported by Rodrigues and Naehrer [41] in a three-year study on the worldwide occurrence of mycotoxins. In North and Central Europe, Southeast Asia, Oceania, and North America, the values were significantly higher (up to 49,000 µg/kg). The concentration of DON and ZEN in the positive cereal grain samples did not exceed EU guidance values of 8000 µg/kg and 2000 µg/kg, respectively, for cereals intended for animal feed [15].

Positive triticale samples were contaminated with all four *Alternaria* toxins and two *Fusarium* toxins (DON and ZEN), with TeA (65%) and DON (58%) being the most abundant. Triticale was reported as the most contaminated grain by Bryła et al. [48], who investigated the presence of *Fusarium* toxins in various grains in Poland. Triticale is a cereal obtained by crossing wheat and rye to improve production. For a long time, it was more resistant than wheat or rye, but its health is steadily declining [49,50]. As there are only few data available on resistance to mould infections and the induction of mycotoxins in triticale, no exact conclusion can be drawn. Furthermore, the production of triticale in Europe increased by up to 90.4% in 2001–2016 [51,52], so that the effects on human and animal health should be considered in the future.

It is difficult to derive trends in mycotoxin contamination because there are several factors that cause annual variations in results, such as climatic influences during planting, growing, and harvesting, as well as exposure to fungal spores, fungal growth, weather conditions, crop management, and storage conditions (e.g., humidity and temperature) [2,8,11]. These data were not collected in the study and therefore no conclusions can be drawn about the correlation of the contamination with *Fusarium* and *Alternaria* toxins on geographical and climatic conditions. However, the available data from the meteorological stations show that the annual average temperatures in all years (2014–2016) were above the long-term average (1981–2010) in 2014, by more than 1.6 °C. The fluctuations in annual average temperatures depend on both the year and the region. Annual precipitation in 2014–2016 fluctuated significantly compared to the long-term average (1981–2010), both by year and by individual regions of Slovenia. In 2014, there was significantly more precipitation (from 5% to more than 40% more) than the long-term average (1981–2010). In 2016, some regions also received more precipitation than the long-term average, but the deviation was slightly smaller (0%–20%); 2015 was drier than the long-term average in most regions. As mentioned above, there are no specific regulations for any of the *Alternaria* toxins in animal feed or food in the European Union. To refine the exposure assessment, representative occurrence data on *Alternaria* toxins in food and feed across European countries are required [1,16].

## 4. Conclusions

This survey study presents important data on the presence of *Alternaria* toxins and other toxins in grains used in Slovenia. To date, in Slovenia, there have been no data on the prevalence of *Alternaria* toxins. The results presented in this work indicate the high prevalence of *Alternaria* and other toxins in cereals in Slovenia. Furthermore, a significantly positive correlation between *Alternaria* toxin with DON and ZEN concentrations was found in the survey. The results showed that regular preventive measures for reducing contamination with *Alternaria* toxins cannot be omitted. Since EFSA [1,16] recommended generating more analytical data on the occurrence of *Alternaria* toxins, more surveys are suggested to reduce contamination by *Alternaria* species and the concomitant hazardous effects on animal and human health.

## 5. Materials and Methods

### 5.1. Sample Collection

Six types of cereal grains (wheat, barley, triticale, rye, oats, and spelt) were collected in all the regions of Slovenia in 2014–2016 to provide country representative samples. The sampling procedure was carried out in accordance with Commission Regulation (EC) No 152/2009 [53]. A total of 433 samples were taken and analysed for the presence of mycotoxins. There were 181 samples of wheat, 107 samples of barley, 81 samples of triticale, 31 samples of rye, 18 samples of spelt, and 15 samples of oats collected over a period of three years. The samples were tested for *Alternaria* toxins (AOH, AME, TeA, and TEN) and for the most relevant mycotoxins (DON, 3-AcDON, 15-AcDON, DAS, HT-2, T-2, OTA, FB1, FB2, and ZEN).

### 5.2. Standards and Chemicals

A mixed trichothecene standard solution in acetonitrile (DON, 3-AcDON, 15-AcDON, T-2, HT-2, DAS), produced by Trilogy (Washington, MO, USA) and individual standards of TeA, TEN, AOH, AME, ZEN, OTA, FB1, and FB2 (Romer Labs, Tulln, Austria) were used. The stock standard solutions and the working standard solutions were prepared in acetonitrile and stored in amber glass vials at –20 °C. The concentrations of the standard stock solutions were 100 µg/mL (DON, 3-AcDON, 15-AcDON, T-2, HT-2, DAS, TeA, TEN, AOH, AME, and ZEN), 50 µg/mL (FB1 and FB2), and 10 µg/mL (OTA). Acetonitrile, methanol (Honeywell, Seelze, Germany), acetic acid (Sigma-Aldrich, Steinheim, Germany), and ammonium acetate (Merck, Darmstadt, Germany) were of p.a. or LC–MS purity. Deionized water was prepared with Milli-Q system (Millipore, Bedford, MA, USA). The extraction solution was a mixture of acetonitrile and deionized water in the ratio 84:16 (*v*/*v*) with the addition of acetic acid (0.5%). The final sample solution was a mixture of deionized water and methanol at a ratio of 80:20 (*v*/*v*). The mobile phase of components A and B were deionized water and methanol, both containing 0.2 g ammonium acetate and 5 mL acetic acid per litre.

### 5.3. Analytical Procedure

For the determination of mycotoxins, a method described in detail by Topi et al. [31] was applied. The procedure consisted of the extraction of mycotoxins from ground cereal samples and the determination with LC–MS/MS and was based on the analytical methods of Rasmussen et al. [54], Lattanzio et al. [55], and Schenzel et al. [56]. The samples were ground to a particle size of 1 mm with a laboratory mill (Retsch ZM 100, Haan, Germany). A sample of 10 g was extracted with 100 mL of a mixture of acetonitrile and deionized water (84:16, *v/v*) for 1 h using a digital linear shaker IKA HS 501 (IKA Labortechnik, Staufen, Germany). A total of 4 mL of the filtered extract was evaporated to dryness under a vacuum using a Syncore Polyvap system (Büchi, Flawil, Switzerland). The dry residue was reconstituted in 0.5 mL of a methanol–water mixture (20:80, *v/v*). An aliquot of 10 µL was injected into the LC–MS/MS system (Acquity UPLC system), coupled to a triple quadrupole mass spectrometer Xevo TQ (Waters, Milford, MA, USA) equipped with an interface for electrospray ionization (ESI) and MassLynx software for data acquisition and processing (Waters, Milford, MA, USA). Specific LC–MS/MS parameters related to specific mycotoxins (retention times, ionization mode, and monitored transitions) are listed in Table 3.

### 5.4. Method Validation

The method validation was carried out with different types of feed. For linearity studies, matrix-matched calibration curves were established by spiking blank samples with calibration standards within the concentration range, as shown in Table 1. The recovery rates were determined by analysing mycotoxin spiked blank feed material and compound feed samples. DON, 3-AcDON, 15-AcDON, T-2, HT-2, DAS, FB1, FB2, and OTA were spiked at LOQ, 2LOQ, and 20LOQ, while *Alternaria* toxins were spiked at LOQ, 4LOQ, and 40LOQ. The spiked concentrations for DON, 3-AcDON, 15-AcDON, T-2, HT-2, and DAS were 40, 80, and 800 µg/kg; for ZEN 20, 40, and 400 µg/kg; for FB1 and FB2 50, 100, and 1000 µg/kg, and for OTA 10, 20, and 200 µg/kg. The *Alternaria* toxins (TeA, TEN, AME, and AOH) were spiked at 12.5, 50, and 500 µg/kg. Spiked samples were prepared in duplicate. The repeatability and within-laboratory reproducibility were expressed as the relative standard deviation (RSD_r_ and RSD_wR_, respectively). The limit of detection (LOD) and limit of quantification (LOQ) of each mycotoxin were estimated as concentrations, resulting in signal-to-noise ratios of 3:1 and 10:1, respectively. However, the lowest successfully validated level was chosen as the limit of quantification (LOQ) for each mycotoxin (Table 1).

### 5.5. Statistical Evaluation

The data on the presence of all detected mycotoxins are left-censored. Therefore, for statistical analysis, the results for mycotoxin concentration below the LOQ were replaced by half of the limit of quantification. The analysis was performed using R Statistical Software (Foundation for Statistical Computing, Vienna, Austria). The differences in the occurrence of mycotoxins between years and cereals were tested with the Kruskal‒Wallis rank test and a multiple comparison test. We compared the occurrence of mycotoxins between cereals with sufficiently large samples: wheat, barley, and triticale. The correlations between the occurrences of mycotoxins were calculated using the Spearman correlation coefficient and adjusted *p*-values (Holm’s method). For all tests, *p* < 0.05 was considered statistically significant.

## Figures and Tables

**Table 1:** Limit of detection (LOD), limit of quantification (LOQ), and linearity data for the analysed mycotoxins.

Mycotoxin	LOD (µg/kg)	LOQ (µg/kg)	Concentration Range (µg/kg)	Correlation Coefficient
DON	12	40	40–800	0.9914
3-AcDON	12	40	40–800	0.9994
15-AcDON	12	40	40–800	0.9934
DAS	12	40	40–800	0.9964
HT-2	12	40	40–800	0.9928
T-2	12	40	40–800	0.9965
OTA	2	5	5–100	0.9965
ZEN	6	20	20–400	0.9938
FB1	15	50	50–1000	0.9992
FB2	15	50	50–1000	0.9984
TEN	4	12.5	12.5–500	0.9965
TeA	4	12.5	12.5–500	0.9982
AOH	4	12.5	12.5–500	0.9970
AME	4	12.5	12.5–500	0.9964

DON: deoxynivalenol; 3-AcDON: 3-acetyldeoxynivalenol; 15-AcDON: 15-acetyldeoxynivalenol; DAS: diacetoxyscirpenol; OTA: ochratoxin A; ZEN: zearalenone; FB1: fumonisin B_1_; FB2: fumonisin B_2_; TeA: tenuazonic acid; TEN: tentoxin; AOH: alternariol; AME: alternariol monomethyl ether.

**Table 2:** Performance characteristics of the analytical procedure for the determination of the analysed mycotoxins.

Mycotoxin	RSD_r_ (%)	RSD_wR_ (%)			Recovery (%)
		CL1 (µg/kg)	CL2 (µg/kg)	CL3 (µg/kg)	
DON	18	25	26	22	95
3-AcDON	7	11	8	7	98
15-AcDON	14	24	19	4	90
DAS	16	24	21	2	97
HT-2	11	22	20	7	104
T-2	3	16	16	14	101
OTA	10	28	20	20	98
ZEN	11	21	21	11	110
FB1	5	13	11	18	97
FB2	9	21	22	19	98
TEN	8	21	9	9	100
TeA	13	22	17	11	99
AOH	14	16	12	9	103
AME	7	15	11	20	102

RSD_r_: relative standard deviation obtained under repeatability conditions (%); RSD_wR_: relative standard deviation obtained under within-laboratory reproducibility conditions (%); CL: concentration level (µg/kg). CL1/CL2/CL3: 40/80/800 µg/kg for DON, 3-AcDON, 15-AcDON, DAS, HT-2, T-2; 50/100/1000 µg/kg for FB1, FB2; 20/40/400 µg/kg for ZEN; 10/20/200 µg/kg for OTA; 12.5/50/500 µg/kg for TeA, TEN, AOH, and AME.

**Table 3:** LC–MS/MS parameters for the analysed mycotoxins.

Mycotoxin	Ionization Mode	RT * (min)	Precursor Ion (m/z)	Quantifier Ion (m/z)	Qualifier Ion (m/z)	Cone Voltage (V)	Collision Energy (V)
DON	ESI+	3.14	297.3	203.1	249.1	18/18	16/10
3-AcDON	ESI+	5.00	339.1	203.1	137.0	24/14	16/5
15-AcDON	ESI+	5.00	339.1	136.95	261.1	16/16	12/10
TeA	ESI-	6.92	196.1	139.0	112.0	44/44	20/24
DAS	ESI+	7.06	384.3	307.2	247.2	20/20	14/10
HT-2	ESI+	8.91	442.4	215.1	263.2	12/12	16/14
AOH	ESI+	9.26	259.1	127.9	185.0	45/15	30/5
TEN	ESI+	9.94	415.3	312.2	199.1	24/24	20/14
FB1	ESI+	10.20	722.4	334.2	352.2	30/30	40/35
T-2	ESI+	10.27	484.4	185.1	215.2	18/18	24/20
OTA	ESI+	11.10	404.2	221.0	239.0	24/24	36/24
ZEN	ESI−	11.30	317.2	131.0	174.95	35/40	22/24
AME	ESI−	12.36	271.0	256.0	228.0	50/50	5/5
FB2	ESI+	12.70	706.4	318.2	336.2	35/35	40/40

* retention time.

## Data Availability

The data presented in this study are available on request from the corresponding author.

## Supplementary Materials

The following are available online at https://www.mdpi.com/article/10.3390/toxins13050304/s1, Table S1: The occurrence of *Alternaria* and *Fusarium* mycotoxins by year. Table S2: The occurrence of *Alternaria* and *Fusarium* mycotoxins in cereals.
