# Supplementary material for: Occurrence of Alternaria and Other Toxins in Cereal Grains Intended for Animal Feeding Collected in Slovenia: A Three-Year Study
Source: Toxins (Basel). 2021 Apr 24;13(5):304. doi: 10.3390/toxins13050304 (PMC8145452; doi:10.3390/toxins13050304)
Supplement: Supplementary file 1 [file toxins-13-00304-s001.zip › toxins-1182075 SU layout.pdf]

Supplementary Materials: Occurrence of Alternaria and Other Toxins in Cereal Grains Intended for Animal Feeding Collected in Slovenia: A Three-Year Study

Janja Babič, Gabrijela Tavčar-Kalcher, Franci Aco Celar, Katarina Kos, Tanja Knific and Breda Jako-vac-Strajn

Table S1. The occurrence of *Alternaria* and *Fusarium* mycotoxins by year.

Yearly Descriptive Statistics	TeA	TEN	AOH	AME	DON	3- and 15-AcDON	ZEN	Total
2014								
No. of samples	173	173	173	173	173	173	173	173
No. of positive samples	67	5	20	21	42	0	14	93
Incidence rate (%)	39	3	12	12	24	0	8	54
Mean (µg/kg)	106	23	113	124	440	–	97	343
Median (µg/kg)	59	21	45	39	299	–	61	139
5th–95th percentile (µg/kg)	15–378	17–33	14–207	13–389	109–1340	–	36–278	17–1338
Max (µg/kg)	666	35	1289	995	1860	–	300	1860
2015								
No. of samples	124	124	124	124	124	124	124	124
No. of positive samples	22	22	20	3	43	8	2	71
Incidence rate (%)	18	18	16	2	35	6	2	57
Mean (µg/kg)	168	25	105	411	846	148	251	643
Median (µg/kg)	52	25	42	78	604	89	251	296
5th–95th percentile (µg/kg)	14–889	15–37	16–295	39–1017	125–3204	46–392	239–262	22–2390
Max (µg/kg)	1053	42	959	1121	4082	491	263	4082
2016								
No. of samples	136	136	136	136	136	136	136	136
No. of positive samples	23	9	8	3	52	0	6	69
Incidence rate (%)	17	7	6	2	38	0	4	51
Mean (µg/kg)	359	34	383	54	666	–	101	682
Median (µg/kg)	219	20	84	31	398	–	89	431
5th–95th percentile (µg/kg)	33–937	15–94	25–1492	27–98	105–2391	–	62–149	30–2459
Max (µg/kg)	2277	116	1836	105	3504	–	149	3504
Total (2014–2016)								
No. of samples	433	433	433	433	433	433	433	433
No. of positive samples (%)	112	36	48	27	137	8	22	231
Incidence rate (%)	26	8	11	6	32	2	5	53
Mean (µg/kg)	170	27	155	148	653	148	112	535
Median (µg/kg)	78	24	44	39	363	89	78	275
5th–95th percentile (µg/kg)	16–558	15–47	14–922	15–813	110–2174	46–392	40–266	20–2098
Max (µg/kg)	2277	116	1836	1121	4082	436	300	4082

No.: number; Mean: the arithmetic mean of the positive samples; Median: the median concentration of the positive samples; Max: the maximum of the positive samples. DAS, HT-2, T-2, FB1 and FB2, and OTA are not included in table. They were detected in a single sample or not detected at all.

Table S2. The occurrence of *Alternaria* and *Fusarium* mycotoxins in cereals.

Cereal Group	TeA	TEN	AOH	AME	DON	3- and 15-AcDON	ZEN	Total
Wheat								
No. of samples	181	181	181	181	181	181	181	181
No. of positive samples	11	4	14	7	62	4	9	79
Incidence rate (%)	6	2	8	4	34	2	5	44
Mean (µg/kg)	42	28	39	34	705	97	77	584
Median (µg/kg)	24	20	35	38	387	69	74	278
5th–95th percentile (µg/kg)	16–109	14–55	14–73	13–60	112–2145	46–189	35–132	23–2091
Max (µg/kg)	154	61	78	69	4082	207	149	4082
Barley								
No. of samples	107	107	107	107	107	107	107	107
No. of positive samples	13	0	4	0	18	0	3	32
Incidence rate (%)	12	0	4	0	17	0	3	30
Mean (µg/kg)	187	–	31	–	521	–	181	390
Median (µg/kg)	37	–	33	–	329	–	238	134
5th–95th percentile (µg/kg)	14–971	–	16–44	–	100–1542	–	60–263	15–1236
Max (µg/kg)	1053	–	44	–	1573	–	266	1573
Triticale								
No. of samples	81	81	81	81	81	81	81	81
No. of positive samples	41	21	8	3	36	0	5	63
Incidence rate (%)	51	26	10	4	44	0	6	78
Mean (µg/kg)	101	26	58	52	784	–	147	544
Median (µg/kg)	78	25	46	23	471	–	147	335
5th–95th percentile (µg/kg)	18–286	15–37	13–131	19–106	156–2585	–	54–271	21–1818
Max (µg/kg)	397	42	150	115	3720	–	300	3720
Rye								
No. of samples	31	31	31	31	31	31	31	31
No. of positive samples	25	8	12	5	14	0	4	29
Incidence rate (%)	81	26	39	16	45	0	13	94
Mean (µg/kg)	181	20	75	29	288	–	58	345
Median (µg/kg)	132	18	82	28	204	–	61	239
5th–95th percentile (µg/kg)	27–507	15–31	27–115	25–34	115–546	–	42–70	39–892
Max (µg/kg)	666	35	116	35	564	–	71	666
Spelt								
No. of samples	18	18	18	18	18	18	18	18
No. of positive samples	10	1	5	8	6	2	0	17
Incidence rate (%)	56	6	28	44	33	11	0	94
Mean (µg/kg)	497	116	569	248	652	324	–	851
Median (µg/kg)	203	116	99	105	180	324	–	427
5th–95th percentile (µg/kg)	34–1692	116–116	21–1640	48–783	117–2368	173–474	–	97–2708
Max (µg/kg)	2277	116	1836	995	3084	491	–	3084
Oats								
No. of samples	15	15	15	15	15	15	15	15
No. of positive samples	12	12	5	4	1	2	1	13
Incidence rate (%)	80	80	33	27	7	13	7	87
Mean (µg/kg)	210	23	514	372	264	75	263	562
Median (µg/kg)	178	23	260	171	264	75	263	297
5th–95th percentile (µg/kg)	24–474	19–26	23–1223	35–992	264–264	61–88	263-263	23–1972
Max (µg/kg)	545	26	1289	1121	264	90	263	1289
Total								
No. of samples	433	433	433	433	433	433	433	433
No. of positive samples	112	36	48	27	137	8	22	231
Incidence rate (%)	26	8	21	6	32	2	5	53
Mean (µg/kg)	170	27	155	148	653	148	112	535
Median (µg/kg)	78	24	44	39	363	89	78	275
5th–95th percentile (µg/kg)	16–558	15–47	14–922	15–813	110–2174	46–392	40–266	20–2098
Max (µg/kg)	2277	116	1836	1121	4082	436	300	4082

No.: number; Mean: the arithmetic mean of the positive samples; Median: the median concentration of the positive samples; Max: the maximum concentration of the positive samples.
